# Improving Photocatalytic Performance Using Nanopillars and Micropillars

**DOI:** 10.3390/ma14020299

**Published:** 2021-01-08

**Authors:** Jessica L. Waite, Julianna Hunt, Haifeng Ji

**Affiliations:** Department of Chemistry, Drexel University, Philadelphia, PA 19104, USA; jlw478@drexel.edu (J.L.W.); julianna.louise.hunt@drexel.edu (J.H.)

**Keywords:** nanopillars, photocatalyst

## Abstract

A recent research emphasis has been placed on the development of highly crystallized nanostructures as a useful technology for many photocatalytic applications. With the unique construction of semiconductor transition metal oxide nanostructures in the form of nanopillars—artificially designed pillar-shaped structures grouped together in lattice-type arrays—the surface area for photocatalytic potential is increased and further enhanced through the introduction of dopants. This short review summarizes the work on improving the efficiency of photocatalyst nanopillars through increased surface area and doping within the applications of water splitting, removal of organic pollutants from the environment, photoswitching, soot oxidation, and photothermalization.

## 1. Introduction

Nanostructures of semiconductive photocatalysts, such as titanium dioxide (TiO_2_), including nanopillars, quantum nanowires, and thin films have been developed for visible light-driven photocatalytic reactions [1]. Metal nanoparticles have been proven to enhance photocatalytic activity due to their surface plasmon resonance properties [2]. Due to the large bandgap of TiO_2_, as well as the low recombination rate of electron–hole pairs and large potential for energy conversion observed by TiO_2_ nanocomposite semiconductors, the wavelength of solar absorption window within these materials is broadened, thus increasing photoactivity [3].

Increased surface area in these nanostructures demonstrated enhanced photoactivity [4]. Specific surface area has shown to be increased by introducing transitional metal oxide nanostructure “guests” doped into the host’s interlayers [5]. With increased surface area, TiO_2_ nanopillars are subject to doping and sensitizing with semiconductors, transition metal ions, and nonmetals with enhancing sustainable energy pathways such as the photoelectrochemical splitting of water into hydrogen, soot oxidation, and conversion of atmospheric carbon dioxide into hydrocarbon structures. A catalyst having a high specific surface area can increase the reaction rate due to an increased number of organic molecules on the surface of the catalyst [5]. Concerning photocatalyst, an increased surface area may create more active sites available for water and hydroxyl absorption, trapping photogenerated charges for radicals to promote photodegradation [5]. These new active sites lead to the formation of other active structures.

Other transition metal oxides are used for water splitting and decomposition of organic pollutants due to their commercial benefits and low environmental risks [6]. Reactive hydroxyl radicals on the surface of titanium dioxide are the key to the catalyst’s water purification abilities. As UV light enters the material, electron–hole pairs are photogenerated to interact in redox reactions at the surface of the catalyst [6]. The electron–hole pairs can dissociate into hydrogen and hydroxyl radicals, which are then used for the decomposition of pollutant levels in water [7].

The efforts of various groups have been compiled for analysis to determine how the photocatalytic efficiency of the nanomaterials has been enhanced. An emphasis is put on the results rather than the synthesis, allowing the reader to see the direct effect of each group’s experimental work.

## 2. Nanopillars for Water Splitting—Improving the Efficiency due to Doping Silicon or Titanium Dioxide Nanopillars

Water splitting using solar energy is of particular interest. Water splitting processes have been used to produce reusable energy sources such as molecular oxygen and hydrogen, contributing to the decline of carbon dioxide emission and dependence on fossil fuels [8]. This photocatalytic process has, therefore, been the subject of many studies due to its valuable products, which can also be produced using photoelectrode in oxygen or hydrogen-evolution reactions. In an electrode used for water splitting, the location of optical absorbance is wherever the electron hole charge carrier can contribute to the surface reactions [4]. The surface, therefore, controls the intake of photons being absorbed on the semiconductor electrode; increased light available to the surface provides more photons to the electrode. Nanopillared surfaces provide such a capability.

Several transition metal oxides have been used for water splitting purposed due to their commercial benefits and low environmental risks [9]. By attaching one-dimensional nanostructures to the surface of such oxides, it is possible to increase the light absorbance by increase surface area.

### 2.1. Titanium Dioxide—TiO_2_

Transitional metal oxides are commonly used as photocatalysts. Titanium dioxide (TiO_2_) has been discovered as an efficient photocatalyst in the reduction in water to produce $H_{2}$ evolution in a very stable, nontoxic, and cost-efficient way. However, the material proves to be problematic in the visible light range and show poor productivity due to a wide band gaps around ~3 eV [10]. Thus, doping TiO_2_ with other materials, such as semiconductors; transition metal ions; and nonmetals, have been studied to improve the photocatalytic efficiency, as introducing other materials with smaller band gaps will improve the productivity of the large band gap material.

One of the most effective approaches to extending the titanium dioxide light absorption range is the doping of titanium dioxide with nonmetal elements. Xing et al. reported a one-step thermal protection method to synthesize an S-doped porous anatase titanium dioxide nanopillar, which maintains its anatase structure up to 700 °C, resulting in high levels of crystallinity and photocatalytic activity in the solar region [11]. The band gap of the pure TiO_2_ sample was found to be 3.2 eV, greater than the band gaps of the four S-doped samples at differing calcination temperature, T-500, T-600, T-700, and T-800, which have band gaps at 2.85, 2.92, 3.00, and 3.16, respectively. This suggests narrowing of the bandgap due to the introduction of S particles, which promotes visible-light absorption and enhanced photon utilization. The H_2_ evolution of the S-doped sample reached 163.9 ${\mu molh}^{-}{}^{1}g^{-}{}^{1}$ for sample T-700, suggesting that a calcination temperature of 700 °C is most efficient for hydrogen evolution. Samples T-500, T-600, and T-800 had H_2_ evolution values of 35.09, 77.39, and 44.79 ${\mu molh}^{-}{}^{1}g^{-}{}^{1}$, respectively. There is no report of hydrogen evolution values for a pure TiO_2_ samples, but it can be implied the study is focused on how calcination temperature of TiO_2_ samples doped with S affects photocatalytic activity.

Qu et al. [11] reported a one-pot method without surfactant for the synthesis of silver-titanium dioxide (Ag–TiO_2_) heterojunction nanopillars for the conversion of solar energy to $H_{2}$. Such nanopillars provide a heterogeneous junction interface when light-harvesting semiconductors and cocatalyst are paired, which improves electron charge transfer and separation, allowing for the conception of a high-performance photocatalyst. The enhancement of light production at this interface is attributed to the surface plasmon resonance properties of metal nanoparticles, such as silver, within the synthesized metal-semiconductor photocatalyst. Three different samples were produced having differing molar ratios of Ag to TiO_2_. An increase in Ag to TiO_2_ ratio shows increased intensity, suggesting higher amounts of Ag particles increase the production of Ag-TiO_2_ composites; an Ag to TiO_2_ molar ratio of 20:1 is the most efficient. This AT-20 (Ag-TiO_2_ at a molar ratio of 20) shows a higher efficiency that is 72.1 μmol$h^{-}{}^{1}$ greater than that of AT-10 and AT-40, whose irradiation values under visible light were 38.2 and 44.2 μmol$h^{-}{}^{1}$, respectively. Compared to a planar sample of Pt-loaded TiO_2_ having irradiation values of 5.4 μmol$h^{-}{}^{1}$, all three samples showed greater photocatalytic performance due to the junctions between Ag cocatalyst and TiO_2_ semiconductor. Therefore, AT-20 was the most efficient in $H_{2}$ production, while the planar sample showed the lowest efficiency.

The photoelectrochemical (PEC) efficiency of the oxides may be increased by means of manipulation of the oxide heterostructure, which, in turn, separates the electron–hole pairs and floods the electron–hole pair deficiencies in the material. The photogenerated electron–hole pairs in the 1D shell contribute to the increased photoelectric features when their charges show a moderate separation. TiO_2_ has also been used as a doping material into other photoactive semiconductor, such as 1D silicon nanopillars (SiNP), to improve the effectiveness of the Si. Pavlenko et al. tested the effectiveness of a silicon nanopillar doped or coated with TiO_2_ and zinc oxide (ZnO) heterostructures [8]. Four samples of silicon nanopillars having different metal oxide heterostructure core shells were produced: a silicon nanopillar with ${\mathrm{TiO}}_{2}$ coating (SiNP/TiO_2_), a silicon nanopillar with ZnO coating (SiNP/ZnO), a silicon nanopillar with both ${\mathrm{TiO}}_{2}$ and ZnO coating (SiNP/TiO_2_/ZnO), and a planar comparison sample of SiNP. The result showed that nanopillars with 1D core shells, specifically containing silicon and both ${\mathrm{TiO}}_{2}$ and ZnO, have the ability to improve photocurrent densities up to 60 and 4 times higher than SiNP/TiO_2_ and SiNP/ZnO at the water-splitting potential of 1.23 V vs. RHE (0.63 V vs. Ag/AgCl), respectively. This is due to the photoelectric properties that pertain to the mixture of these oxides. The SiNP/TiO_2_/ZnO sample showed to have higher efficiency in charge separation and transfer. When comparing the plane SiNP sample to those doped with ZnO, TiO_2_, or both metal oxides, there is a significant increase in intensity of the GIXRD spectra when doping is present, changing intensity from >100 a.u. to approximately 1200 a.u. when doped with both metals.

### 2.2. Hematite—Fe_2_O_3_

To optimize the light intake, materials with smaller band gaps that can more efficiently utilize the UV-VIS spectra are used for water splitting. Hematite ($\alpha \text{-}{\mathrm{Fe}}_{2}O_{3}$) is an n-type semiconductor with various uses. Fe_2_O_3_ is commonly used for photoanode in water splitting due to its commercial benefits, but, more importantly, it has a low band gap of 2.2 eV [4] and solar-to-hydrogen efficiency of ~17%, thus hematite works in the visible light range. However, due to hematite’s poor minority charge carrier mobility, the short hole diffusion length and high electron–hole recombination rate of hematite restrict its possibility for application within the photoelectrochemical cell. The production of hematite nanostructures may be the answer to this problem.

**Figure 1:**
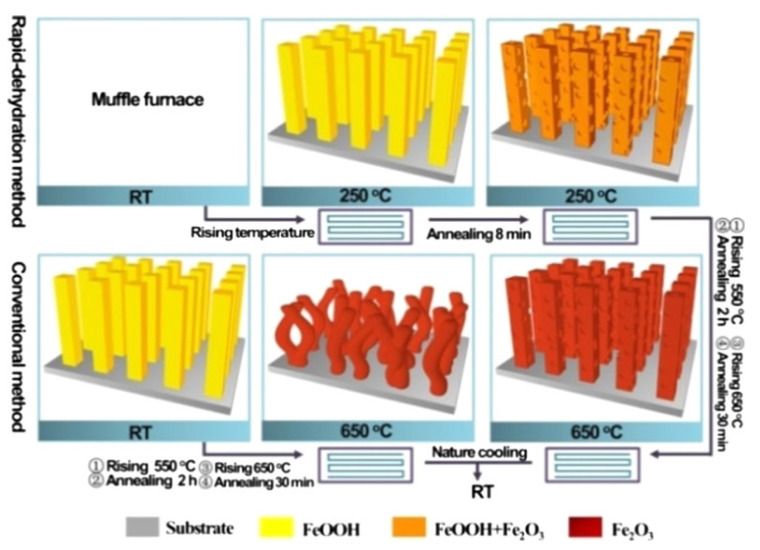
Schematic illustration of the preparation of conventional C-Fe_2_O_3_ compared to rapid dehydration RD-Fe_2_O_3_. (Adapted from [9] Figure 1, with permissions from ACS).

Liao et al. reported a rapid dehydration strategy involving the quasi-topotactic transformation of FeOOH nanorods to porous Fe_2_O_3_ pillars, capable of photoelectrochemical water oxidation. The implementation of Fe_2_O_3_ nanorods would reduce the distance needed for hole diffusion and overcome the poor charge transport limitation [9]. This strategy is accomplished by directly dehydrating the FeOOH nanorods in a high-temperature furnace (Figure 1). Two samples were produced, including a sample of nanorod a conventional temperature sample, C–Fe_2_O_3_ (the planar ${\mathrm{Fe}}_{2}O_{3}$ sample with conventional temperature-rising technique) and a Rapid Dehydration sample, RD-Fe_2_O_3_. Photocurrent density was found to have improved by 270%, with values of 2.0 mA/cm^2^ (1.23 V vs. RHE) and 3.5 mA/cm^2^ (1.71 V vs. photoanodes RHE) for the RD-Fe_2_O_3_ photoanode, compared to the conventional C-Fe_2_O_3_ sample having photocurrent density values of 0.75 mA/cm^2^ (1.23 V vs. RHE) and 1.48 mA/cm^2^ (1.71 V vs. photoanodes RHE). This can be accredited to the lowered chare-carrier recombination (electron-hole pair recombination rate) due to single crystalline nanopillars, increase light harvesting ability due to the production of longer nanopillars, and a porous structure that shortens the distance between bulk material and the interface between electrode and electrolyte.

**Figure 2:**
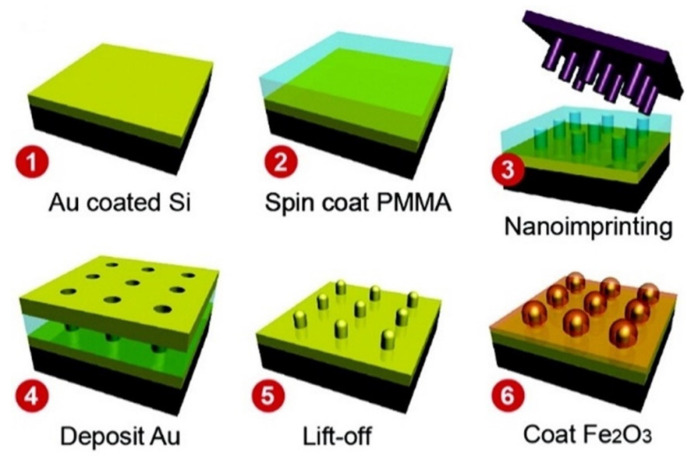
Silicon semiconductor is coated with gold. The nanoprinting technique is then displayed to show how the material is coated with Au nanopillar arrays (AuNPAs) and then layered with Fe_2_O_3_. (Adapted from [4] Figure 1, with permissions from ACS).

Gao et al. [4] created samples of gold photoanodes having Au nanopillar arrays (AuNPAs) for use as electrical contacts and plasmonic couplers with hopes of increasing the photoelectric activity of the Hematite iron (III) oxide photoanodes. Synthesis of the samples can be seen in Figure 2. An increase in surface area of 30% in the electrode of the AuNPAs is observed compared to the planar sample. A photocurrent enhancement of about 40% was observed in the patterned AuNPA sample over the planar sample at 1.5 V vs. RHE (reversible hydrogen electrode), and it can be increased to 50% by increasing the thickness of the AuNPAs.

Ahn et al. investigated the applicability of the hematite iron oxide species in water splitting performance by synthesizing a hematite and Ti-doped SiO_x_ passivation layer structure (Ti–SiO_x_/np-Fe_2_O_3_) to be compared to a simple Ti–Fe_2_O_3_ sample. The thin passivation layer of Ti-doped of amorphous SiO_x_ was synthesized in situ by hydrothermal/annealing processes to induce nanopores and increase photoelectrochemical performance [12]. The surface area of the Ti–(SiO_x_/np-Fe_2_O_3_) sample increased by 2.5 times compared to the simple Ti–Fe_2_O_3_ sample. The Ti-(SiO_x_/np-SiO_x_) sample showed a photocurrent density of 2.44 mA cm^-2^ at 1.23 V_RHE_, a 200% enhancement of the simple Ti–Fe_2_O_3_ sample having a photocurrent density of 1.23 mA cm^−2^ at 1.01 V_RHE_. The high photocurrent density results obtained for the Ti-doped substrate are attributed to the protected surface states and the increased surface area because of its nanosized pore characterization. 

Table 1 compares the performance of the nanomaterials and their planar counterparts for water splitting.

## 3. Nanopillars for Removing Organic Pollutants

Reactive hydroxyl radicals on the surface of photocatalysts such as TiO_2_ are the key to the catalyst’s pollutants-removing abilities. As UV light enters the material, electron–hole pairs are photogenerated to perform redox reactions at the surface of the catalyst. The electron–hole pairs can create or generate hydrogen and hydroxyl radicals, which are then used for the decomposition of pollutant levels in water.

### 3.1. Improving the Efficiency of Oxides due to Larger Surface Area

To produce thin film materials with more capable photocatalytic abilities, it has been questioned how the photocatalytic properties of oxides may be manipulated towards into a porous or nanopillared morphology. The synthesis of materials exhibiting characteristics such as a nanopillars structure, mesoporous network, efficient adsorption capacity, and increased surface area are of high interest in the field of photocatalysis.

The application of nonsilica containing materials onto electric, photonic, and magnetic nanodevices suggests a solution for increased photocatalytic properties in place of previously used materials. Titanium dioxide is commonly used for water purification due to its commercial benefits as well as low corrosion rates. The common anatase phase (3.2 eV) of titanium dioxide shows the highest rates of photocatalytic activity due to high electron functioning. The morphology and pore/particle size have significant influence over the photocatalytic processes of the TiO_2_. A reduction in the size of the TiO_2_ photocatalyst allows for greater surface area for redox reactions to take place.

Kimura et al. investigated porous anatase thin films produced due to the deformation of 3D mesostructures to achieve a better understanding of the photocatalytic properties based on crystallization and porosity [13]. The thin film samples being investigated included a 3D hexagonal mesostructured titania thin film prepared with a precursor solution, a 3D mesostructured film, and a commercial anatase film. Degradation of methylene blue (MB) was observed in each sample. Crystallinity is seen completely in both hexagonal and mesostructured samples, therefore, surface area and pore size are investigated to determine photocatalytic abilities. S_eff-MB_ values show information about photocatalytic abilities relative to effective surface area of the MB. S_eff-MB_ value of the hexagonal structure was reported at 0.0095 $\mathrm{mol}/{\mathrm{cm}}^{3},$ compared to mesostructure’s value of 0.0148 $\mathrm{mol}/{\mathrm{cm}}^{3}$, and the commercial anatase sample’s value of 0.0016 $\mathrm{mol}/{\mathrm{cm}}^{3}$. The commercial comparison sample showed significantly decreased photocatalytic abilities due to low porosity. Porous films of fully crystallized anatase nanoparticles show enhanced photocatalytic abilities and the deformation of 3D mesostructures allow for proper adjustment of intercrystallite mesospaces. This method can be utilized to produce electrodes with increased porous structures in nanodevices as well as the production of enhanced photocatalyst systems.

Tin (IV) oxide (SnO_2_) is a conducting oxide with lower conduction band than TiO_2_. Cheng et al. reported that the photocatalytic properties can be enhanced by incorporating a SnO_2_/TiO_2_ core-shell nanopillar-array structure that utilizes both the heterojunction and high surface of SnO_2_/TiO_2_ [3]. The resulting products were tested with methylene blue (MB) irradiation. TiO_2_ films with and without a SnO_2_ interlayer were produced, having decay constants of 0.094 and 0.065 $h^{-}{}^{1}$. It was found that SnO_2_ nanopillars act as a sink for electron carriers released from TiO_2_ and increases the lifetime of hole carriers for methylene blue (MB) degradation. The photocatalytic properties were improved by 180% and 300% for sample with and without SnO_2_ interlayer when compared to a planar TiO_2_.

### 3.2. Degradation of Organic Pollutants due to Photocatalytically Enhanced TiO_2_ Nanostructures

The prevalence of unwanted organic micropollutants in pharmaceuticals, drinking water, and personal care products raises a serious concern to the environment and the public health domain. Some of them are highly toxic chemical species even at very low concentrations. The exploration of heterojunction nanopillars has been accomplished through the addition of sulfide, nitride, and oxide cocatalysts onto TiO_2_ semiconductor surface to create a light-harvesting interface. By enhancing periodical texture through a micropillar array of TiO_2_ on flat surface materials, as well as exploring structural imperfections through band gap structure alteration at boundary defect sites, the photoactivity of TiO_2_ semiconductor nanostructures improves.

Steroidal hormones, specifically those known as endocrine disrupting chemicals (EDCs) are highly dangerous to the environment due to threatening levels of estrogen and low ability to be biologically decomposed. These toxic chemicals may damage human puberty rates and spermatozoa levels as they enter the ecosystem through runoff and excretion, whether it be direct or due to commercial use. More specifically, an EDC known as 17α-Ethynylestradiol, or EE2, is one such dangerous hormone used in birth control pills and hormone therapies. Water ways and sewage lines are contaminated with the pollutant, leading to increased exposure. Commonly used semiconductor TiO_2_ offers a way for heterogeneous photocatalytic processes to clean the pollutants from water systems. Electron–hole pairs of the titanium dioxide allow for the absorption of pollutants through oxidation and reduction reactions, resulting in a superoxide radical, $O_{2}^{-}$, and hydroxyl radical, OH· ($E^{0}=2.80\;V$). In water systems where EE2 was present, the hydroxyl radical removed the pollutant by 92%. Photocatalytic degradation of indicators such as Alizarin Yellow further indicate the strength of the photocatalytic abilities [8]. Recently, two thin film samples were prepared to investigate how nanopillars surface affect the degradation of EE2: a titanium dioxide nanopillar thin film produced with PEG filler (S2), and a thin film produced without PEG filler (S1). The sample using only UV light is used as the “planar” reference sample. Percent degradation at a pH of 6 shows about 16% removal using UV light, whereas samples S1 and S2 have degradation percentages around 20% and 30%, respectively. Compared to solely UV light treatment, samples S1 and S2 proved to be efficient treatments for organic pollutant water cleansing.

**Figure 3:**
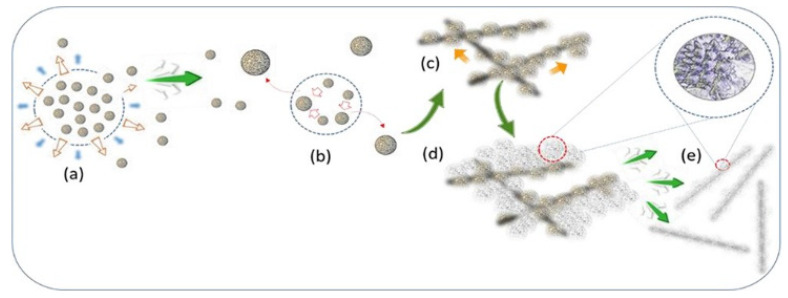
Schematic representation showing formation of surface-modified quantum wires (SMoQWs) from nanoclustered nanowires (NCNWs). (**a**) Centers of nucleation form and (**b**) assemble into larger particles by the process of coalescence driven by Ostwald ripening and undergo (**c**) multidirectional growth into NCNWs and (**d**) removal of high-energy weakly bonded particles to form (**e**) nanopillars of SMoQWs. (Adapted from [14] Figure 1, with permissions from ACS).

At the semiconductor surface level, the lattice defects influence the electron-transfer processes of semiconductor TiO_2_ species and affect the photocatalytic properties of the semiconductor at these interfacial boundary sites through the introduction of dopants. Additionally, altering the band gap structure at boundary defect sites through chemical intervention techniques, such as imidazolium type ionic liquids, allow for more efficient charge transfer routes and an increased oxygen evolution for semiconductor nanowires. Song et al. [14] explored the case of TiO_2_ in which the imidazolium ionic liquid semiconductor (liquid–solid) interface is developed as a tuning surface for altering shape and size of TiO_2_ nanocrystals into aggregated clusters—this alteration tunes the defect sites at boundary regions and allows for the development of efficient photocatalytic materials. Surface-modified quantum wires (SMoQWs) were observed for photocatalysis as well as the zero-dimensional nanoclustered nanowires (NCNWs) from which they were fabricated. The transition from NCNWs (associated with the TiO_2_ rutile phase) to SMoQWs occurs because of nitrogen doping of intercrystalline void spaces (Figure 3). When exposed directly to UV light, the SMoQWs samples had a 15.8% increase in photocatalytic properties compared to NCNWs. The SMoQWs sample showed degradation rates 8 times faster than that of NCNWs. By engineering void defects in crystal structures of TiO_2_ nanopillars, low-dimensional quantum material growth from nanoclusters generates heterocoordination sites and enhances photocatalytic performance.

### 3.3. Improving the Efficiency with Decorated TiO_2_ Nanopillars

The use of TiO_2_ as a degradation agent for organic pollutants as well as water splitting is of high interest, as previously mentioned in various studies due to the compound’s photocatalytic properties. TiO_2_ is commonly used for its photocatalytic properties, yet its use is faulted by low quantum yield and low utilization rates of the visible light spectrum (~5%). The deposition of noble metals on TiO_2_ can alter the surface properties of TiO_2_ and inhibit recombination carriers, therefore changing the photocatalytic activity.

**Figure 4:**
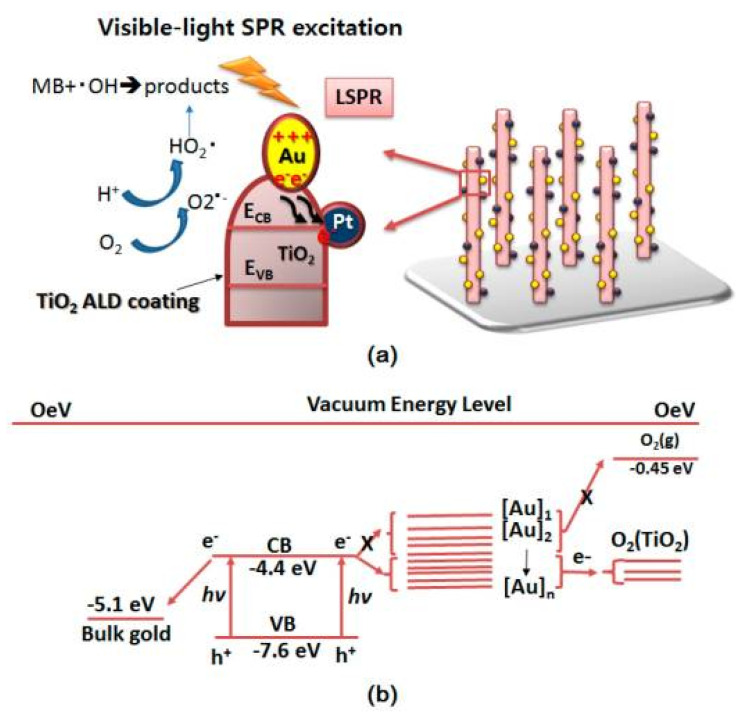
(**a**) The charge transfer process of TiO_2_ ALD/Au/Pt/TiO_2_ NPAs under UV-VIS lights; (**b**) Fermi level of nanoscale gold particles. (Adapted from [15] Figure 6, with permissions from MDPI’s open access license).

Shuang et al. investigated the codecorated Au/Pt noble metal nanoparticles on TiO_2_ vertically aligned nanopillar arrays to measure organic pollutant (dye) degradation [15]. As one quantum size effect approach to enhancing the accessibility of TiO_2_ as a photocatalytic semiconductor, TiO_2_ nanopillar arrays were vertically synthesized on substrates in order to maximize reaction surface area. The metallic nanoparticles were applied through atomic layer deposition, and the TiO_2_ was annealed at three different temperatures to ensure the photocatalytic performance of the nanopillar. In studying the photocatalytic mechanism (Figure 4), the noble metal nanoparticles observe a “hot” electron shift from the surface of the Au to the Pt cocatalyst through the TiO_2_ conduction band. The photoelectrochemical performance and the dye degradation of the vertically aligned nanopillar array were measured at various annealing temperatures; the highest experimental dye degradation efficiency was reported as 75.3% at 300 °C, while the efficiency for Au/Pt–TiO_2_ without atomic layer deposition (ALD) was 49.3%. Conclusively, this ALD method for synthesis of Au/Pt–TiO_2_ with annealing represents a rational design for the functionality of vertically aligned nanopillars to efficiently maximize solar energy usage for photocatalytic dye degradation of common organic pollutants.

Shuang et al. also introduced Au and Pt nanoparticles (NPs) on TiO_2_ nanopillar arrays (NPAs) to increase the photocatalytic properties of the TiO_2_ for pollutant degradation [2]. In their study, they used metal coupling techniques to improve photocatalysis by the Schottky barrier conduction band (CB) electron trapping and increase electron–hole pair lifetime. The use of metals slows the charge-pair recombination by acting as an electron sink or by trapping electrons on the CB to be transferred to electron acceptors. This study produced four samples of NPs decorated with TiO_2_ composite NPAs: pure TiO_2_ NPAs, Pt–TiO_2_ NPAs, Au–TiO_2_ NPAs, and Au/Pt–TiO_2_ NPAs. The resulting Au/Pt NPs decorated with TiO_2_ composite NPAs show highly enhanced photocatalytic activity for degradation of methylene orange (MO) and overall photoelectric (PEC) activity in comparison to other NPAs. The photocatalytic efficiency is 21 times greater under UV-VIS light and 13 times higher under visible light than that of pure TiO_2_ due to the electron-sink system of Pt and surface plasmon resonance of Au nanopillars.

Standard water treatment systems are unable to rid waterways of such antibiotics pollutants, such as tetracycline, creating a need for oxidation processes including TiO_2_ photocatalyst [16]. Tiwari et al. aimed to improve the limited photocatalytic functions of TiO_2_ through the doping of noble metal nanoparticles of Au onto the titanium dioxide. These nanoparticles show surface plasmon resonance effect under UV and visible light irradiation and act as cocatalyst in separation of electron–hole pairs, aiding the reactions taking place at the surface of the material. Two samples were produced both containing gold doping with or without a poly(ethylene glycol) (PEG) solid template, known as Au/TiO_2_ (A) and Au/TiO_2_ (B). Sample (A) was nontemplated without PEG solid, and sample (B) was templated with PEG solid. The tetracycline was mineralized by the photocatalytic degradation under UV-A light (λ > 330 nm). Repeated use of the thin films was possible due to its stability. The degradation of the tetracycline pollutant was mainly due to the hydroxyl radicle produced at the valence and the conduction bands of the semiconductor. Results showed that samples containing Au particles show much greater photocatalysis. It is suggested that use of the PEG solid template increases stability and photocatalytic abilities compared to sample (A). In final analysis, the embellishment of noble metals onto titanium dioxide photocatalyst increase the degradation rates of micropollutants in water systems.

Nitrogen dopants have been used to attempt to alter the bandgap of TiO_2_ [17]. This study conducted by Jiang et al. hoped to fabricate TiO_2_ nanopillar arrays doped with nitrogen that have adjustable bandgaps of varying wavelengths within the visible light range. Ag nanoparticles were also embedded onto TiO_x_N_y_ nanopillars by photoreduction in Ag^+^ (aq) under irradiation of visible light. TiN films were oxidated to produce TiO_x_N_y,_ which was compared to the unoxidized TiN. Photoactivity was tested at different oxidation temperatures. A temperature of 550 °C shows the greatest photocatalytic ability as Rhodamine B (RhB) was degraded >50% and a color change was observed. Incorporation of Ag particles significantly increases photocatalytic properties. The TiO_x_N_y_ matrix bandgap was found to be adjustable, and the addition of Ag particles embedded into the matrix enhances the photocatalytic abilities.

Zhang et al. used cesium titanate to produce pillared nanocomposites. Cesium titanate has similar properties to TiO_2_ and has a large band gap. Transition metal doping has been used to increase light absorption due to band gap narrowing. The hybridization of two different semiconductors were known to decrease the electron–hole pair recombination probability [5]. The semiconductor guest particles are inserted into the interlayer of the 2D semiconductor host lattice to create a heterojunction structure. Therefore, the samples being investigated included an Fe-doped cesium titanate (having two semiconductors to act as guest particles and a host lattice, ${\mathrm{Cs}}_{0.68+x}{\mathrm{Ti}}_{1.83-x}{\mathrm{Fe}}_{x}O_{4}$) and ZnO pillared doped titanate (only having one semiconductor for band gap narrowing, $\mathrm{ZnO}/{\mathrm{Ti}}_{1.83-x}{\mathrm{Fe}}_{x}O_{4}$). A bare ZnO nanoparticle was used for comparison. The bare ZnO nanoparticles degraded methylene blue (MB) by 4% after a 90-min irradiation. During the same increment of irradiation, the ${\mathrm{Cs}}_{0.68+x}{\mathrm{Ti}}_{1.83-x}{\mathrm{Fe}}_{x}O_{4}$ sample degraded 25.8% of MB molecules (x = 0.04) and the $\mathrm{ZnO}/{\mathrm{Ti}}_{1.83-x}{\mathrm{Fe}}_{x}O_{4}$ sample had a maximum degradation rate of 67.7% (x = 0.02). Surface area increased from the cesium sample (1 $m^{2}/g$) to the ZnO nanoparticle (12 $m^{2}/g$) to the ZnO pillared doped titanate (103 $m^{2}/g$). Degradation of MB increased from the ZnO nanoparticle to the ${\mathrm{Cs}}_{0.68+x}{\mathrm{Ti}}_{1.83-x}{\mathrm{Fe}}_{x}O_{4}$ sample to the $\mathrm{ZnO}/{\mathrm{Ti}}_{1.83-x}{\mathrm{Fe}}_{x}O_{4}$ sample. All the samples were compared to those not containing Fe doping, and it was concluded that iron ions increased the response to the UV range.

### 3.4. Improving the Efficiency with Decorated Hematite Nanopillars

Shuang et al. studied the photocatalytic performance of CdS nanoparticle decorated with hematite nanopillar arrays (CdS NP/α-Fe_2_O_3_ NPAs) and compared that with a pure hematite nanopillar array sample [18]. Electron–hole separation can be achieved using CdS, which has a small bandgap and high absorption rate. Photogenerated electrons from CdS nanoparticles transfer into the hematite to achieve charge-carrier separation. The photocurrent density and degradation efficiency for NPA’s with CdS NP and without was about 94% degradation in 75 min vs. 78% dye degradation, respectively. The CdS NP/α-Fe_2_O_3_ NPAs showed increased photocurrent density of 2.0 mA${\mathrm{cm}}^{2}$ at 0.4 V vs. Ag/AgCl. The result showed that heterojunctions of CdS hematite provides efficient solar energy conversion and photocatalysis. 

Table 2 compares the performance of the nanopillars and their planar counterparts for removing the organic pollutants.

## 4. Nanopillars for Breaking Organic Chemicals for Analysis

Degradation of organic pollutants can be used in analysis. As an example, drug candidates can often form toxic metabolites. Taking place in the liver, phase I of drug metabolism involves enzymes, which biotransform xenobiotics into polar metabolites. It is in this phase that toxic metabolites are formed and therefore is of particular interest for research. Phase I oxidation reactions can be mimicked or modeled by electrochemistry techniques, such as liquid chromatography and mass spectrometry taking place in an electrochemical cell [19]. To mimic phase I of metabolism of organic compounds, a TiO_2_-coated microchip was developed using titanium dioxide nanoreactors and UV radiation for production, detection, and identification purposes of metabolites and reaction products. The microchip can be used to produce, detect, and identify photocatalytic reaction products of specific drugs that may mimic phase I of metabolism. The TiO_2_ microchip proved to have faster speed and higher sensitivity compared to other in vitro models. The microchip model shows greater photocatalysis ability opposed to degradation efficiency. Moreover, 50% of the maximum signal of verapamil metabolites were reached in 2 min. A correlation was found between metabolites found in vitro and in vivo methods compared to reaction products seen on the microchip. Further information about change in degradation rates and photocurrent density was not given. It was concluded that the titanium dioxide microchip can predict phase I metabolites in the early stages of drug study and can help uncover new drug contenders.

**Figure 5:**
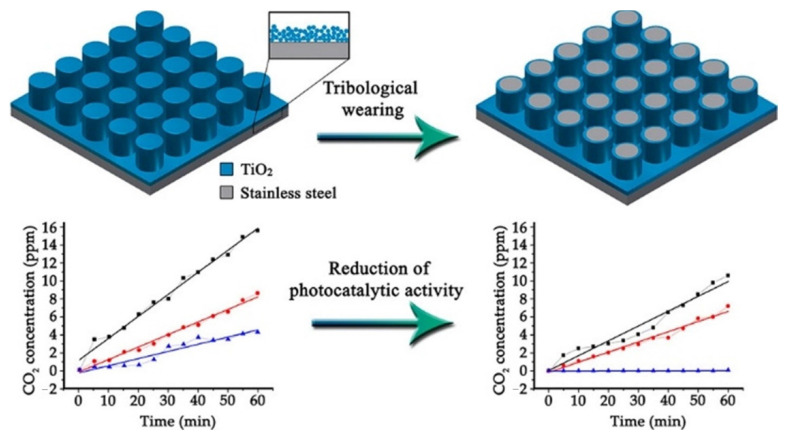
Schematic representation of TiO_2_ on stainless steel micropillars (top), measured photocatalytic activity of three prepared samples for 1 h (bottom left), and measured photocatalytic activity of three samples after tribological wear (bottom right). (Adapted from [20] Figure 1, with permissions from Elsevier).

In another work, Temerov et al. explore the liquid flame spray (LFS) technique of TiO_2_ nanoparticles onto stainless steel while measuring deviations in photocatalytic activity and comparing the efficiency of flat surface nanostructures to the metal injected molded (MIM) stainless steel array structures [20]. The study reported the increased active surface area of the MIM manufactured micropillar arrays after LFS with TiO_2_ on stainless steel, resulting in a higher photocatalytic activity and a resistance to tribological wear (Figure 5). In comparison, the flat surface reference sample lost complete photocatalytic activity as a result of harsh condition exposure; the flat sample reported a low photocatalytic activity of 4.2 ppm/h, while the high density micropillars reported an activity of 15.6 ppm/h. MIM micropillar arrays produced on stainless steel surfaces increase surface area and increase photocatalytic activity after liquid flame spray deposition of titanium dioxide nanoparticles. Decrease in surface area and resulting photocatalytic performance after tribological wear was observed at 100% decrease, 32% decrease, and 16.2% decrease for the sample without micropillars, high-density micropillars, and low-density micropillars, respectively. A mixture of rutile and anatase can show more enhanced photocatalytic activity compared to the singular anatase phase. The incorporation of TiO_2_ nanoparticle application to stainless steel micropillars through the LFS technique with MIM microtextures provides a highly photocatalytic and versatile technique for light-harvesting that can withstand mechanical and environmental wear.

## 5. Nanopillars for Photoswitching Achieved by Varying Surface Hydrophobicity and Hydrophilicity

**Figure 6:**
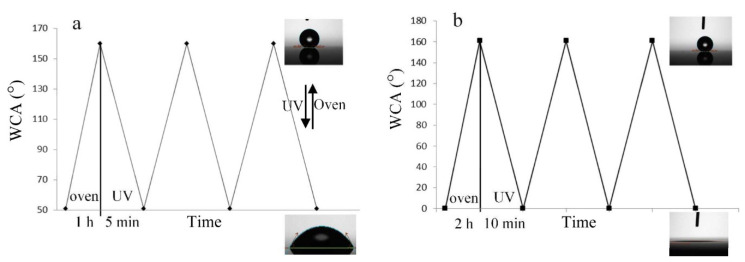
(**a**) Hydrophilic to superhydrophobic transitions by 1 h annealing in oven at 60 °C and 5 min of UV exposure; (**b**) superhydrophilic to superhydrophobic transitions by 2 h annealing in oven at 60 °C and 10 min of UV exposure. (Adapted from [21] Figure 4, with permissions from ACS).

The concept of controlling surface wettability and adhesion of the superhydrophobic surfaces of inorganic oxides has become highly advantageous in biosensing, self-cleaning, solar cells, and liquid–liquid extraction applications. The controlled wettability of these material surfaces influences photoresponsivity because of transitions between the bistable states of inorganic oxides including titanium dioxide, zinc oxide, and tungsten oxide. As a result of UV light exposure, the hole diffusion rate to titanium dioxide surface increases, leaving the titanium–oxygen bonds within the lattice extremely weak and available for increased interaction and hydration with water molecules, thus increasing surface hydrophilicity. Hoshian et al. report the results of three photo/thermal switching processes after measuring the titanium dioxide micropillar overhang geometry and surface nanostructuring while controlling UV exposure time [21]. The test samples include a flat substrate, simple micropillars, micropillars with overhand, flat nanostructured surface, simple micropillar with nanostructures, and a micro/nanostructured overhang sample. The results regarding the three photoswitching processes of rolling to sticky superhydrophobic states, superhydrophobic to hydrophilic states, and superhydrophobic to superhydrophilic states after 1, 5, and 10 min of UV exposure yield slow switching speeds, which remain impractical for applications (Figure 6). The reported photoswitching properties of titanium dioxide indicate that reversible switching from rolling to sticky superhydrophobic states requires only 1 min of UV exposure, due to the photoactive titanium dioxide thin film and overhang geometry of the nanopillars. Quantitative data were not provided in comparison of the samples. The application of TiO_2_ thin film and overhang geometry micropillars presents a novel approach to speeding the wetting transition from rolling to sticky superhydrophobic states and improving photoswitching properties.

## 6. Nanopillars for Photocatalytic Soot Oxidation

**Figure 7:**
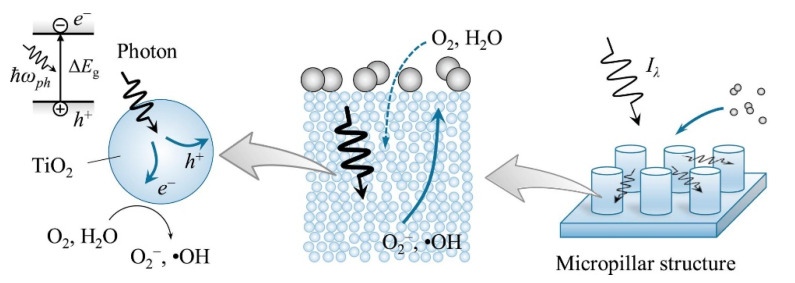
Schematic of the multiscale phenomena occurring in the photocatalytic soot oxidation on TiO_2_ microstructured substrate. (Adapted from [22] with permissions from Elsevier).

The generation of TiO_2_ radicals capable of oxidizing soot pollutant particles has been confirmed. Recently, Kameya et al. explored the effect of microstructure of TiO_2_ on soot oxidation rate by measuring photon absorption of plain substrate (Figure 7) at varying thicknesses and micropillar substrate [22]. The study reports nonlinear increase in electron–hole generation, photon absorption, and soot oxidation rate as a function of increasing plain TiO_2_ substrate thickness (<2.5 μm). Two samples were produced for comparison, both TiO_2_ microstructures on plain or micropillared substrates. Additionally, the incorporation of TiO_2_ nanopillars enhanced photon absorption through multiple scattering, resulting in increased rates of soot removal/oxidation with increasing substrate thickness. The application of TiO_2_ nanopillar substrate, as well as instituting increased film thickness, presents an efficient alternative for environmental repair of soot removal from air particles.

## 7. Nanopillars for Photothermalization

The harvesting of high energy and nonequilibrium “hot” electrons represents a recent application of metal nanostructures with the focus of analyzing photocatalytic activity and the correspondence of electronic structural and optical properties within specific metals.

**Figure 8:**
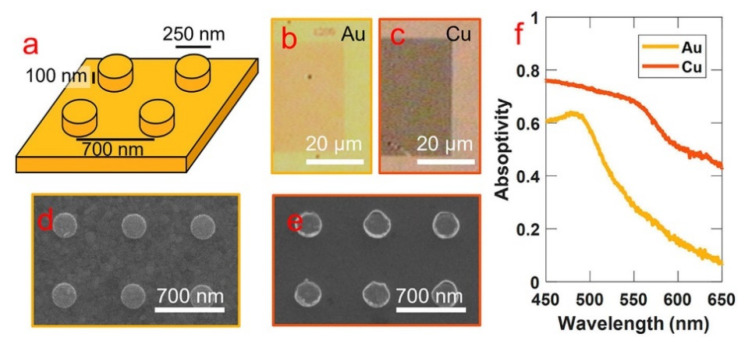
(**a**) A schematic of the fabricated nanostructure with a pitch of 700 nm, a height of 100 nm, and a cylinder diameter of 250 nm on top of a thin film with thickness of 150 nm. Optical and SEM images of the nanostructure are shown in (**b**,**d**) for gold and (**c**,**e**) for copper, respectively. (**f**) The absorptivity of the two nanostructures. (Reproduced from [23] Figure 1, with permissions from AIP).

Hogan and Sheldon [23] investigated the photothermalization dynamics of copper “hot” electrons during continuous wave (CW) optical excitation. Two samples for comparison are gold (Au) and copper (Cu) nanostructures with differing hot electron temperature and lifespan measurements. They reported a considerable abundance, high reactivity, and large steady state population of “hot” electrons in copper, compared to gold and silver (Figure 8). The implementation of copper, rather than gold, metal nanostructures presents a powerful application for plasmonic “hot” electrons in enhancing photocatalytic and photochemical performance, as a result of the characteristically large population of “hot” electrons and the decreased electron lifetime in copper. The kinetic energy measurement of these “hot” electrons provides insight to the photochemical properties of metals, especially copper, silver, and gold; this plasmonic system is applicable to the process of water splitting, carbon dioxide reduction, and ammonia generation.

## 8. Nanopillars for Conversion of Carbon Dioxide to Energy—Increasing Photosynthetic Efficiency through Doping of InP Nanopillars with TiO_2_

**Figure 9:**
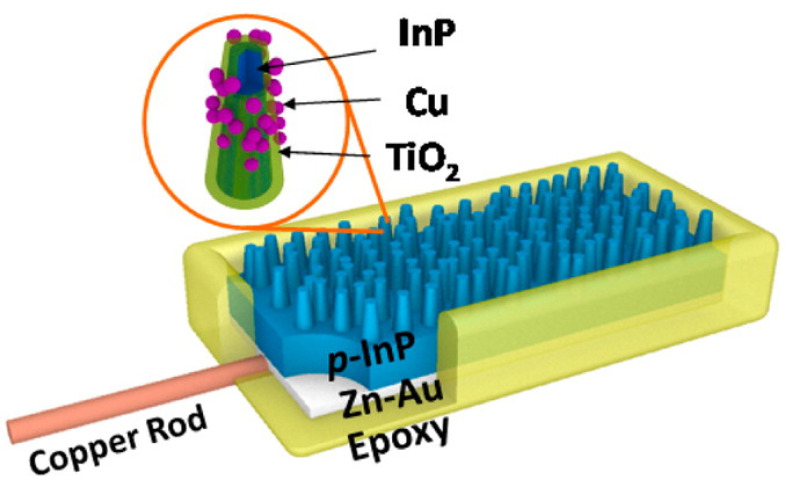
Schematic diagram of TiO_2_-passivated InP nanopillars with Cu cocatalyst nanoparticles. (Adapted from [24] Figure 1, with permissions from ACS).

As an environmentally conscious area of study receiving recent attention in response to the increasing levels of carbon dioxide in the atmosphere, the artificial synthesis technique converting carbon dioxide into hydrocarbon structures, such as methanol and formaldehyde, with semiconductor nanopillars has gained traction. The optimal band gap region of the semiconductor required for photoreduction is reported as 1.2–1.4 eV, with the additional necessity for proximity of electron affinity of semiconductor and carbon dioxide redox potential. For effective photoreduction, the surface recombination rate of photon-induced electron–hole pairs must remain low [24]. Qiu et al. utilized TiO_2_-passivated Tin- Phosphide (InP) nanopillars to investigate carbon dioxide reduction to methanol under 532 nm illumination within aqueous medium; the study measures methanol production as a function of InP sample preparation—with and without the titanium dioxide-passivated layers and copper cocatalyst nanoparticles (Figure 9)—using high resolution transmission electron microscopy (HRTEM). They reported enhancement of photoconversion efficiency following the incorporation of oxygen vacancies in titanium dioxide by atomic layer deposition; The application of TiO_2_-doped InP nanopillars in carbon dioxide reduction showed an increased Faraday efficiency (8.7%) of methanol production and allows for an 8-fold increase in the performance of this artificial photosynthetic pathway.

## 9. Conclusions

In summary, this review analyzed the improvement of photocatalytic performance of vertical nanopillar arrays of a variety of photocatalysts, including catalyst in the presence of metal and nonmetal dopants and lattice defects at catalytic interfaces.

Applications of photocatalyst nanopillars have been considered in the context of water splitting, removing organic pollutants from atmospheric and aqueous environments, photoswitching of hydrophobic and hydrophilic surfaces, and energy harnessing through carbon dioxide catabolism; an overall improvement to nanopillar photocatalytic efficiency is achieved through doping with metals and nonmetals, increasing surface area with nanopillar and nanowire structures and introducing lattice defects at catalytic interfaces.

## Figures and Tables

**Table 1:** The performance of the nanomaterials and their planar counterparts.

Nanomaterial for Enhanced Photocatalytic Performance	Planar Comparison Sample	Improved Photocatalysis Compared to Planar Sample
S-doped TiO_2_ with hydrogenation evolution value of 163.9 ${\mu molh}^{-}{}^{1}g^{-}{}^{1}$ at a calcination temperature of 700 °C. [10]	No quantitative comparison to pure TiO_2_, but when compared to S-doped samples calcinated at 500, 600, and 800 °C, H_2_ evolution values are significantly lower: 35.09, 77.39, and 44.79 ${\mu molh}^{-}{}^{1}g^{-}{}^{1}$.	N/A
AT-20: 72.1 ${\mu molh}^{-}{}^{1}$ greater AT-10: 38.2 ${\mu molh}^{-}{}^{1}$ AT-40: 44.2 ${\mu molh}^{-}{}^{1}$ [11]	Pt-loaded TiO_2_: irradiation values of 5.4 ${\mu \mathrm{molh}}^{-}{}^{1}$	Raman spectra values increase from 147.7 ${\mathrm{cm}}^{-}{}^{1}$ for TiO_2_ comparison to 152.7 ${\mathrm{cm}}^{-}{}^{1}$ for Ag-TiO_2_ nanopillars.
SiNP/TiO_2_ SiNP/ZnO SiNP/TiO_2_/ZnO [8]	SiNP	SiNP/TiO_2_/ZnO shows photocurrent densities 60 times higher than SiNP/TiO_2_ and 4 times higher than SiNP/ZnO. An increase in intensity from 100 to 1200 a.u. is found compared to the planar SiNP sample.
Rapid dehydration sample RD-${\mathrm{Fe}}_{2}O_{3}$: 2.0 mA/cm^2^ (1.23 V vs. RHE) and 3.5 mA/cm^2^ (1.71 V vs. photoanodes RHE) [9]	Conventional temperature sample $C\text{–}{\mathrm{Fe}}_{2}O_{3}$: 0.75 mA/cm^2^ (1.23 V vs. RHE) and 1.48 mA/cm^2^ 157 (1.71 V vs. photoanodes RHE).	Photocurrent density improved by 270%.
Patterned AuNPAs [4]	Planar Au substrate	30% surface area increase; 50% photocurrent enhancement over solar spectrum.
Ti-doped SiO_x_ photocurrent density of 2.44 mA cm^−2^ at 1.23 V_RHE_, [12]	Ti-Fe_2_O_3_: photocurrent density of 1.23 mA ${\mathrm{cm}}^{-}{}^{2}$ at 1.01 V_RHE_	Surface area enhancement of 2.5 times compared to planar sample, 200% photocurrent density enhancement.

**Table 2:** The performance of the nanopillars and their planar counterparts.

Nanopillar for Removing Organic Pollutants	Comparison Sample	Overall Improved Efficiency Compared to Planar Sample
3D hexagonal mesostructured titania thin film 3D mesostructured film [13]	Commercial anatase film	Surface area is compared through Seff-MB values. Values were re-ported as 0.0095, 0.0148, and 0.0016 $\mathrm{mol}/{\mathrm{cm}}^{3}$ for the hexagonal mesostructured, mesostructured, and commercial samples, respectively.
SnO_2_/TiO_2_ core-shell nanopillar array [3]	Flat TiO_2_	180% (with SnO_2_) and 300% (without SnO_2_) enhancement in photocatalytic properties compared to flat TiO_2_.
S2 (with PEG filler) S1 (without PEG filler) [8]	UV light treatment	UV light shows 16% degradation, 20% for S1, and 30% for S2.
SMoQW (surface-modified quantum wires) [14]	NCNW (nanoclustered nanowires)	15.8% improvement in photocatalytic performance compared to NCNW
Au/Pt–TiO_2_ with atomic layer deposition [15]	Au/Pt–TiO_2_ without atomic layer deposition	Dye degradation rates of 75.3% and 49.3% for samples with and without ALD, respectively.
Au/Pt–TiO_2_ [2]	Pure TiO_2_ NPA	Au/Pt–TiO_2_ showed an enhanced photocatalytic efficiency of 21 times greater than that of pure TiO_2_ under UV–VIS light, and 13 times greater under visible light.
Au/TiO_2_ (A) (nontemplated without PEG solid) Au/TiO_2_ (B) (templated with PEG solid) [16]	Simple UV-A	Sample Au/TiO_2_-B maximum percent tetracycline degradation of 68% compared to 41% of the simple UV sample.
Oxidized TiO_x_N_y_ [17]	TiN	Approximately 50% degradation compared to plane TiN.
$\mathrm{ZnO}/{\mathrm{Ti}}_{1.83-x}{\mathrm{Fe}}_{x}O_{4}$ ${\mathrm{Cs}}_{0.68+x}{\mathrm{Ti}}_{1.83-x}{\mathrm{Fe}}_{x}O_{4}$ [5]	Bare ZnO	Bare ZnO degraded MB at 4% efficiency, while ${\mathrm{Cs}}_{0.68+x}{\mathrm{Ti}}_{1.83-x}{\mathrm{Fe}}_{x}O_{4}$ had degrading rate of 25.8, and $\mathrm{ZnO}/{\mathrm{Ti}}_{1.83-x}{\mathrm{Fe}}_{x}O_{4}$ showed a degradation rate of 67.7%.
CdS NP/α-Fe_2_O_3_NPAs [18]	Pure hematite NPAs	Dye degradation with and without CdS NP was 94% and 78%, respectively.

## Data Availability

No new data were created or analyzed in this study.
